# Intrafollicular Retinoic Acid Signaling Is Important for Luteinizing Hormone-Induced Oocyte Meiotic Resumption

**DOI:** 10.3390/genes14040946

**Published:** 2023-04-20

**Authors:** Fupeng Wang, Yawen Tang, Yijie Cai, Ran Yang, Zongyu Wang, Xiaodong Wang, Qianying Yang, Wenjing Wang, Jianhui Tian, Lei An

**Affiliations:** State Key Laboratory of Animal Biotech Breeding, National Engineering Laboratory for Animal Breeding, Key Laboratory of Animal Genetics, Breeding and Reproduction of the Ministry of Agriculture and Rural Affairs, College of Animal Science and Technology, China Agricultural University, No. 2 Yuanmingyuan West Road, Beijing 100193, China

**Keywords:** LH, Retinoic acid, EGF, oocyte meiotic resumption, C-natriuretic peptide, zinc finger protein 36

## Abstract

It has been clear that retinoic acid (RA), the most active vitamin A (VA) derivative, plays a central role in governing oocyte meiosis initiation. However, it has not been functionally determined if RA participates in luteinizing hormone (LH)-induced resumption from long-lasting oocyte meiotic arrest, which is essential for haploid oocyte formation. In the present study, using well-established in vivo and in vitro models, we identified that intrafollicular RA signaling is important for normal oocyte meiotic resumption. A mechanistic study indicated that mural granulosa cells (MGCs) are the indispensable follicular compartment for RA-prompted meiotic resumption. Moreover, retinoic acid receptor (RAR) is essential for mediating RA signaling to regulate meiotic resumption. Furthermore, we found zinc finger protein 36 (ZFP36) is the transcriptional target of RAR. Both RA signaling and epidermal growth factor (EGF) signaling were activated in MGCs in response to LH surge, and two intrafollicular signalings cooperate to induce rapid *Zfp36* upregulation and *Nppc* mRNA decrease, which is critical to LH-induced meiotic resumption. These findings extend our understanding of the role of RA in oocyte meiosis: RA not only governs meiotic initiation but also regulates LH-induced meiotic resumption. We also emphasize the importance of LH-induced metabolic changes in MGCs in this process.

## 1. Introduction

Retinoic acid (RA), an active derivative of vitamin A (VA), is involved in a number of functions in vertebrates [1,2]. Among these, RA plays an important role in regulating cell cycle and differentiation, etc. [3], and is essential for regulating meiotic initiation in both male and female germ cells [4]. In female embryonic ovaries, RA triggers germ cells’ initial transition from mitosis to meiosis by stimulating a critical meiotic marker, named stimulated by retinoic acid gene 8 (*Stra8*), which is expressed in embryonic ovaries just before meiotic initiation [5,6]. In addition, increasing in vivo and in vitro evidence indicates that RA also participates in the process of folliculogenesis, oocyte maturation and ovulation [2]. Of interest, a series of previous in vitro studies among species have suggested that RA may promote the resumption of oocyte meiosis [7,8,9,10,11,12], a critical event that is essential for haploid oocyte formation [13], despite the controversial results [14,15]. However, these studies are primarily based on in vitro supplementation assays, and the role of RA in regulating oocyte meiotic resumption has never been systematically confirmed via in vivo physiological analysis and in vitro functional experiments. In addition, most above-mentioned studies haven’t focused on the mechanism by which RA prompts oocyte meiotic resumption, and the underlying mechanism remains unclear.

In mammals, oocytes are arrested at the diplotene stage of meiosis until the surge of luteinizing hormone (LH) restarts meiosis and initiates the ovulatory process. Thus, successful oocyte meiotic resumption is the prerequisite for female fertility. The progression of gonadotropin-controlled oocyte meiosis is tightly regulated by C-natriuretic peptide (CNP). Before the LH surge, CNP is primarily synthesized by mural granulosa cells (MGCs) and binds to NPR2 on cumulus cells (CCs) throughout the follicle to stimulate cGMP production, thus maintaining meiotic arrest [16,17]. Following the LH surge, CNP and its encoding messenger RNA (mRNA) natriuretic peptide precursor C (*Nppc*) decrease immediately and restart meiosis [18]. Our recently published study has shown that TTP, also known as zinc finger protein 36 (ZFP36), a ubiquitously expressed mRNA-destabilizing protein, is the critical mechanism that underlies the LH-induced rapid decrease in *Nppc* mRNA and oocyte meiotic resumption. *Zfp36* mRNA is transiently up-regulated in MGCs in response to the LH surge. Then, upregulated TTP binds to *Nppc* mRNA 3′ UTR and, in turn, degrades *Nppc* mRNA [19].

By reanalyzing a previously published transcriptome that presents the rapid effects of LH on gene expression in the MGCs of mouse preovulatory follicles [20], we found that LH triggered the activation of the retinol metabolism pathway. More importantly, we found several retinoic acid response element (RARE) motifs located in the transcriptional regulatory regions of *Zfp36*. Taken together, these facts imply that RA may participate in LH-induced oocyte meiotic resumption by prompting rapid upregulation of *Zfp36.*

In this study, we systematically confirmed the role of RA in prompting oocyte meiotic resumption using the in vivo VA-deficient mouse model and in vitro culture models, including in vitro cultured preovulatory follicles, in vitro matured cumulus-oocyte complexes (COCs) and denuded oocytes. In addition, focusing on the receptor that mediates RA signaling and its downstream target that regulates meiotic progression, we explored the underlying mechanism by which RA regulates oocyte meiotic resumption. Thus, our study updates the role of RA in regulating the progression of oocyte meiosis: RA is important not only for meiotic entry but also for meiotic resumption.

## 2. Material and Methods

### 2.1. Animals

Eight-week-old ICR female mice were kept in a temperature-controlled room with 12-h alternating light/dark. All experiments with mice were approved by the Institutional Animal Care and Use Committee of China Agricultural University. The ethical clearance approval number is AW11107102-1-2.

Weanling female mice used for in vivo experiments were fed a vitamin A deficient diet (VAD; Beijing HFK Bioscience, Beijing, China; Table S2) for up to 6 weeks.

### 2.2. Culture of Preovulatory Follicles

Female mice were i.p. injected with 5 IU Equine chorionic gonadotropin (eCG; Ningbo Sansheng Biotechnology, Zhejiang, China) to stimulate follicle development to the preovulatory stage, as previous studies reported [21,22,23,24,25]. After 48 h, in vivo oocyte meiotic resumption was induced by an i.p. injection of 5 IU human chorionic gonadotrophin (hCG; Ningbo Sansheng Biotechnology). For in vitro culture, preovulatory follicles (POFs) were microdissected from eCG-primed mouse ovaries with 25-gauge needles under a stereomicroscope in Leibowitz L15 medium (Thermo Fisher Scientific, Waltham, MA, USA) supplemented with 5% fetal bovine serum (FBS; Thermo Fisher Scientific), 100 U Penicillin-Streptomycin (Thermo Fisher Scientific) as previously described [21]. Then they were transferred through α-MEM (Thermo Fisher Scientific) supplemented with 10% FBS, 100 U penicillin G, and 100 μg streptomycin sulfate 3 times before treatment. Subsequently, the POFs (5–30 POFs/group) were cultured for the indicated time in fresh α-MEM supplemented with RA (10 μM), with or without BMS493 (10 μM; Selleck, Houston, TX, USA), under 95% O_2_ and 5% CO_2_. To assess the ratio of in vitro germinal vesicle breakdown (GVBD), POFs were punctured to release the cumulus-oocyte complexes (COCs)and placing them in supplemented α-MEM, oocytes were denuded of cumulus cells and evaluated for morphological evidence of GVBD under a stereomicroscope.

### 2.3. Culture of COCs

COCs were isolated by puncturing the ovaries from eCG-stimulated mice with 25-gauge needles in the supplemented Leibowitz L15 medium. After isolation, COCs with homogeneous cytoplasm and more than 3 layers of unexpanded cumulus cells were selected as good-quality oocytes, as mentioned previously [26,27]. Then COCs were washed in the final incubation medium and cultured for the indicated time. The culture medium was α-MEM supplemented with 3% (wt/vol) bovine serum albumin (BSA; Sigma-Aldrich, Burlington, USA), 100 U Penicillin-Streptomycin, with or without BMS493 (10 μM; Selleck), UVI3003 (10 μM; Selleck). Cultures were maintained in an atmosphere of 95% O_2_ and 5% CO_2_ at 37 °C. The ratio of in vitro germinal vesicle breakdown (GVBD) was assessed after culture and oocytes were denuded of cumulus cells and evaluated for morphological evidence of GVBD under a stereomicroscope which refers to the dissolution of the huge nucleus of an oocyte that is arrested in prophase of meiosis I. The oocyte nucleus presents an irregular envelope surrounding dispersed condensed chromatin and is marked by the extrusion of the first polar body [28,29].

### 2.4. Culture of Mouse MGCs

Ovaries were removed from the female mice, and the MGCs were extracted by puncturing ovaries. Collected MGCs were purified by filtration using a 70-μm cell strainer (BS-70-CS; Beijing Labgic Technology, Beijing, China) centrifuged at 200× *g* for 5 min. After brief centrifugation and washing with DMEM/F-12 medium (Thermo Fisher Scientific) supplemented with 1% FBS, 100 U Penicillin-Streptomycin, MGCs were re-suspended in DMEM/F-12 culture media supplemented with 10% FBS, 100 U Penicillin-Streptomycin in 6-wells plate at 37 °C and 5% CO_2_ overnight to allow the cells to attach. Before treatment, cells were washed thoroughly with phosphate-buffered saline (PBS) to remove non-adherent cells. Subsequently, the cells were cultured in fresh medium supplemented with different final concentrations (0.7 nM, 7 nM, 70 nM, or 700 nM) of RA (Sigma-Aldrich), EGF (20 ng/mL; Sigma-Aldrich), 0.1 μM TTNPB (Selleck), 1 μM BMS493 (Selleck), 0.1 μM PD0325901 (Selleck) or various combinations. Standard conditions were maintained for the control group. After the indicated time, the cells were harvested for further experiments.

### 2.5. RNA Extraction and Quantitative RT-PCR Analysis (qRT-PCR)

The total RNA from in vitro cultured MGCs was isolated using TRIzol (Thermo Fisher Scientific) according to the manufacturer’s protocol. The cDNA synthesis was run in 20 μL volumes containing 1000 ng of total RNA by using the HiScript III RT SuperMix for qPCR (Vazyme Biotech, Nanjing, China) according to the manufacturer’s instructions. For quantitative PCR analysis, 10 ng of cDNA (total RNA equivalent) per well in duplicate was amplified using gene-specific primers in Table S1 and SsoFast EvaGreen Supermix (BioRad Laboratories, CA, USA) on a Bio-Rad CFX96 Real-Time PCR System. The amplification conditions were as follows: initial denaturation for 30 s at 95 °C and 40 cycles of 5 s at 95 °C, annealing and elongation for 5 s at 60 °C. For each gene, at least 3 technical replicates and three biological replicates were assayed. *Gapdh* mRNA was used as a loading control, and data were analyzed using the 2^−ΔΔCt^ method.

### 2.6. Western Blotting

Protein lysates were prepared from collected granulosa cells, and a cell homogenization solution (Beyotime Biotechnology, Beijing, China) containing 1% Halt Protease and Phosphatase Inhibitor Cocktail (Thermo Fisher Scientific) was used to isolate protein from granulosa cells. The supernatant was collected after 10 min centrifugation at 15,000× *g* at 4 °C, and protein was quantified using an Enhanced Bicinchoninic Acid Protein Assay Kit (Beyotime Biotechnology). The samples were denatured in the same volume of 2 × Laemmli sample loading buffer (Bio-Rad Laboratories) with 5% β-mercaptoethanol (Sigma-Aldrich) for 5 min at 100 °C and stored at −80 °C for future use. Approximately 10 μg of total protein sample was loaded in each well for 12% SDS-PAGE and then transferred onto microporous PVDF membranes (Millipore) at 4 °C. Membranes containing the transferred proteins were blocked using 5% milk powder (*w*/*v*) in Tris-buffered saline containing 0.1% Tween 20 (TBST; 20 mM Tris–HCl, 150 mM NaCl and 0.1% Tween 20, pH 7.6) for 2 h at room temperature (RT). Membranes were incubated overnight at 4° C with primary antibodies at pre-determined concentrations (1:200, TTP (A-8), Santa Cruz, Cat#SC-374305; 1:1000, GAPDH, CST, Cat#2118s). Blots were washed in TBST and then incubated in horseradish peroxidase (HRP)–conjugated secondary antibodies (1:5000, Golden Bridge, Beijing, China) for 1 h at RT. After three washes in TBST, blots were developed using enhanced chemiluminescence reagents (Millipore), exposed digitally with Tanon 5200 chemiluminescent imaging system (Tanon, China)

### 2.7. GSEA Analysis

The retinol metabolism, reactome signaling by EGFR, and the response to retinoic acid correlated gene set were downloaded from the Kyoto Encyclopedia of Genes and Genomes (KEGG) databases (KEGG: Kyoto Encyclopedia of Genes and Genomes). Gene Set Enrichment Analysis (GSEA) was performed with these 3 gene sets by using javaGSEA software.

### 2.8. Cleavage under Targets & Release Using Nuclease (CUT&RUN)

CUT&RUN was performed as previously described [30,31,32]. The qRT-PCR primer is shown in Table S1. Retinoic Acid Receptor α (SC 515796, Santa Cruz Biotechnology, CA, USA) antibody was used in this experiment. Granulosa cells were collected and resuspended in 600 μL cold nuclear extraction (NE) buffer (20 mM HEPES-KOH, pH 7.9, 10 mM KCl, 0.5 mM Spermidine, 0.1% Triton X-100, 20% glycerol, freshly added protease inhibitors) for 10 min. 20 μL Concanavalin A beads (Polysciences) were prepared per sample. Beads were washed and resuspended in 300 uL Binding Buffer (20 mM HEPES-KOH, pH 7.9, 10 mM KCl, 1 mM CaCl2, 1 mM MnCl2). After centrifugation, Nuclei were added to beads and incubated for 10 min at room temperature (RT). After nuclei binding, samples were placed on a magnetic stand for 5 min, and the beads were resuspended in 1 mL cold blocking buffer (20 mM HEPES, pH 7.5, 150 mM NaCl, 0.5 mM Spermidine, 0.1% BSA, 2 mM EDTA, freshly added protease inhibitors) for 5 min. Then samples were placed on a magnetic stand and resuspended in 250 uL cold Wash Buffer (20 mM HEPES, pH 7.5, 150 mM NaCl, 0.5 mM Spermidine, 0.1% BSA, freshly added protease inhibitors). The primary antibody was added to a final concentration of 1:100 at 4 °C for 2 h. IgG was used as a negative control. Protein A-micrococcal nuclease (pA-MN; 0.95 ug/uL pA-MN was a gift from Wei Xie’s lab) was added to a final concentration of 1:750 at 4 °C for 1 h. Samples were resuspended in 150 uL cold wash buffer, and 3 uL 100 mM CaCl_2_ was added to the sample for 30 min. The reactions were stopped using 2 X STOP buffer (200 mM NaCl, 20 mM EDTA, 4 mM EGTA, 50 ug/mL RNaseA, 40 ug/mL glycogen, 10 pg/mL yeast spike-in DNA) for 20 min at 37 °C, and 3 uL 10% SDS and 2.5 uL 20 mg/mL Proteinase K were added to the supernatants incubated at 70 °C for 10 min. DNA was purified using phenol/chloroform/isoamyl alcohol (PCI; Ameresco), and the sample could then be used for qRT-PCR analysis.

### 2.9. Reanalysis of Published RNA-Sequencing Data

The original down sequence (Raw Reads) was obtained from the GEO data repository (GSE167939). Raw RNA-seq reads after trimming by Trim-galore (version 0.6.5) were aligned to the mouse (GRCm38/mm10) genome using HISAT2 (version 2.2.1) with default parameters. Raw read counts for each gene were generated using the featureCounts program based on the mouse gene annotation (Mus_musculus.GRCm38.102.chr.gtf). The read counts for each gene were calculated, and the expression values of each gene were normalized by TPM. To identify differentially expressed genes in RNA-seq, the DESeq2 R package was used for differential gene expression analyses with cutoffs FoldChange > 2 and FDR values (P adjusted: Padj values) < 0.05.

### 2.10. Statistical Analysis

Experiments were independently replicated for a minimum of three times unless otherwise specified, and all data is reported as the mean ± standard error of the mean (SEM). Student’s t-test was used to analyze the significant difference between the 2 groups. For multiple comparisons, data were analyzed using a 1-way ANOVA followed by Tukey’s multiple comparison test using IBM SPSS 18. Statistically significant differences were defined as * for *p* < 0.05, ** for *p* < 0.01, and *** for *p* < 0.001.

## 3. Results

### 3.1. VA Deficiency Impairs Oocyte Meiotic Resumption

**Figure 1:**
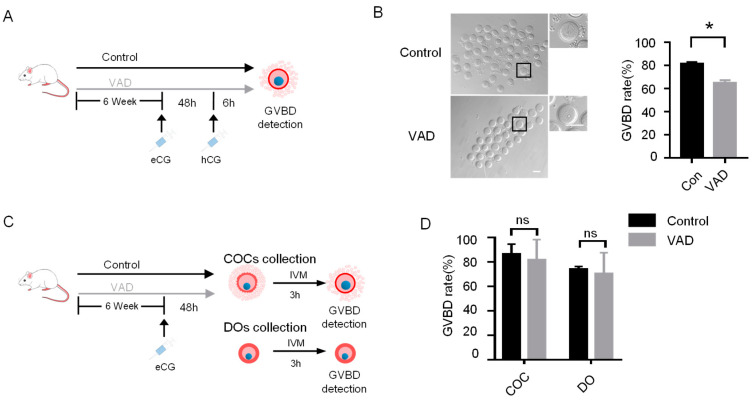
VA deficiency impairs oocyte meiotic resumption. (**A**) Flow chart of experimental design based on in vivo VAD mouse model. (**B**) Quantitative analysis of GVBD rates in control and VAD mouse. * *p* < 0.05. Left panel: representative image of GV and GVBD oocytes from control or VAD mouse. (**C**,**D**) Flow chart of experimental design in vitro maturation (IVM) of COCs and DOs from control and VAD mouse (**C**) and quantitative analysis of GVBD rates when using COCs or DOs. All data are presented as the mean ± SEMs of three independent experiments. ns, not significant (*p* > 0.05). * *p* < 0.05.

To explore the role of RA in oocyte meiotic resumption, we used an in vivo mouse model that was established by feeding a VA-deficient (VAD) diet for up to 6 weeks, which was frequently used to study the physiological functions of RA [2,33,34]. When oocyte meiotic resumption, scored as germinal vesicle breakdown (GVBD) percentage, was evaluated at 6 h post-hCG (Figure 1A), VAD mice showed impaired meiotic resumption compared with that in the wild-type controls (Figure 1B). Next, to determine the follicular compartment responsible for RA-prompted oocyte meiotic resumption, we isolated cumulus-oocyte complexes (COCs) and denuded oocytes (DOs) from preovulatory follicles of stimulated VAD and control mice (Figure 1C). After in vitro maturation (IVM), oocyte meiotic resumption was not affected by VA deficiency when culturing neither COCs nor DOs (Figure 1D). Thus, our in vivo and in vitro results suggest that VA deficiency impairs oocyte meiotic resumption in an MGC-dependent manner.

### 3.2. LH Activates RA Synthetic and Metabolic Pathway in Preovulatory MGCs

**Figure 2:**
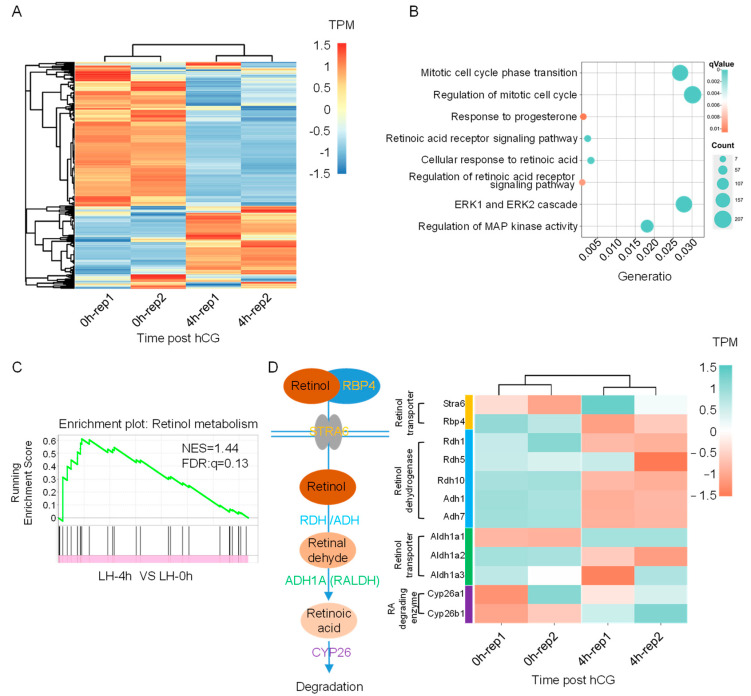
LH activates RA synthetic and metabolic pathways in preovulatory MGCs. Data are presented based on the reanalysis of previously published transcriptome data that present rapid effects of LH on gene expression in the MGCs of mouse preovulatory follicles. (**A**) Heat map of the hierarchical clustering results of DEGs in response to LH surge. (**B**) Bubble plot of the enriched GO terms of DEGs in response to LH. The *X*-axis in the bubble plot represents GeneRatio, while the *y*-axis indicates different biological processes. (**C**) GSEA analysis showing enrichment of Retinol metabolism, Normalized enrichment score (NES) and false discovery rate (FDR) q value are shown. (**D**) Schematic illustration of the retinoic acid metabolic pathway (Left panel) and the heat map of relevant genes in response to LH surge.

Having confirmed VA deficiency impairs oocyte meiotic resumption, we next attempted to ascertain whether RA, the most active VA derivative, is primarily responsible for these findings. We reanalyzed a previously published transcriptome that presents rapid effects of LH on gene expression in the MGCs of mouse preovulatory follicles [20]. Among differentially expressed genes responding to LH surge (Figure 2A) are functionally associated with retinoic acid signaling transduction (Figure 2B). GSEA analysis also indicated that the retinol metabolic pathway was activated in MGCs during LH-induced meiotic resumption (Figure 2C). In detail, genes associated with cellular retinol uptake and RA synthesis were generally upregulated during this process, whereas genes encoding RA-degrading enzymes were downregulated (Figure 2D), suggesting intrafollicular RA signaling may be responsible for VAD-induced impairment of oocyte meiotic resumption.

### 3.3. RA Signaling Disruption Impairs Oocyte Meiotic Resumption

**Figure 3:**
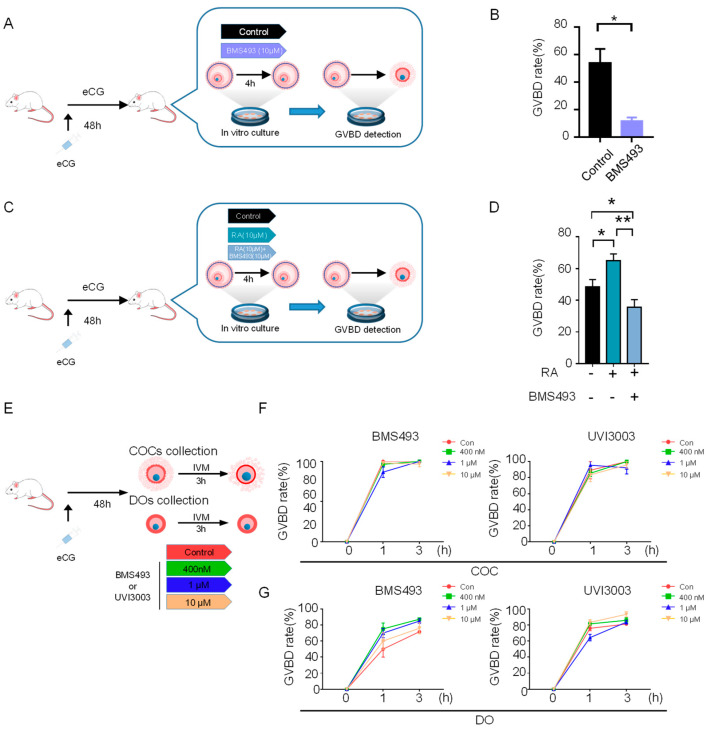
RA signaling disruption impairs oocyte meiotic resumption. (**A**) Flow chart of experimental design based on in vitro culture of eCG-primed preovulatory follicles treated with or without RAR pan-inhibitor BMS493. (**B**) Quantitative analysis of GVBD rates of oocytes collected from preovulatory follicles cultured under different IVM conditions. * *p* < 0.05 (**C**) Flow chart of experimental design based on in vitro culture of eCG-primed preovulatory follicles treated with RA alone or together with BMS493. (**D**) Quantitative analysis of GVBD rates of oocytes collected from preovulatory follicles cultured under different IVM conditions. * *p* < 0.05, ** *p* < 0.01. (**E**) Flow chart of experimental design based on IVM of COCs and DOs groups with or without different concentrations of BMS493 and UVI3003. (**F**,**G**) Quantitative analysis of GVBD rates of oocytes collected from COCs (**F**) and DOs (**G**) cultured with BMS493 or UVI3003. All data are presented as the mean ± SEM of three independent experiments. * *p* < 0.05.

Next, we attempted to provide direct evidence supporting the role of RA signaling in oocyte meiotic resumption. We isolated preovulatory follicles from eCG-primed mouse ovaries. Preovulatory follicles were cultured with LH for 4 h in the presence or absence of a RAR inhibitor, and we detected oocyte meiotic resumption after LH exposure (Figure 3A). We found that blockage of RA signaling by using 10 μM RAR pan-inhibitor BMS493 significantly impaired oocyte meiotic resumption scored as GVBD rate (Figure 3B). In contrast, RA supplementation during the culture significantly facilitated meiotic resumption, and the facilitating effect can be eliminated by RAR inhibition (Figure 3C,D and Figure S1A,B). In addition, in line with results from in vivo VAD model, RA signaling blockage in both in vitro cultured COCs and DOs (Figure 3E) showed that either RAR inhibition by BMS493 (Figure 3F,G, left panel) or RXR inhibition by UVI3003 (Figure 3F,G, right panel), didn’t affect the progression of meiotic resumption. These observations further support the critical role of MGCs in mediating RA-prompted oocyte meiotic resumption.

### 3.4. Zfp36 Is the Potential Transcriptional Target of RAR

**Figure 4:**
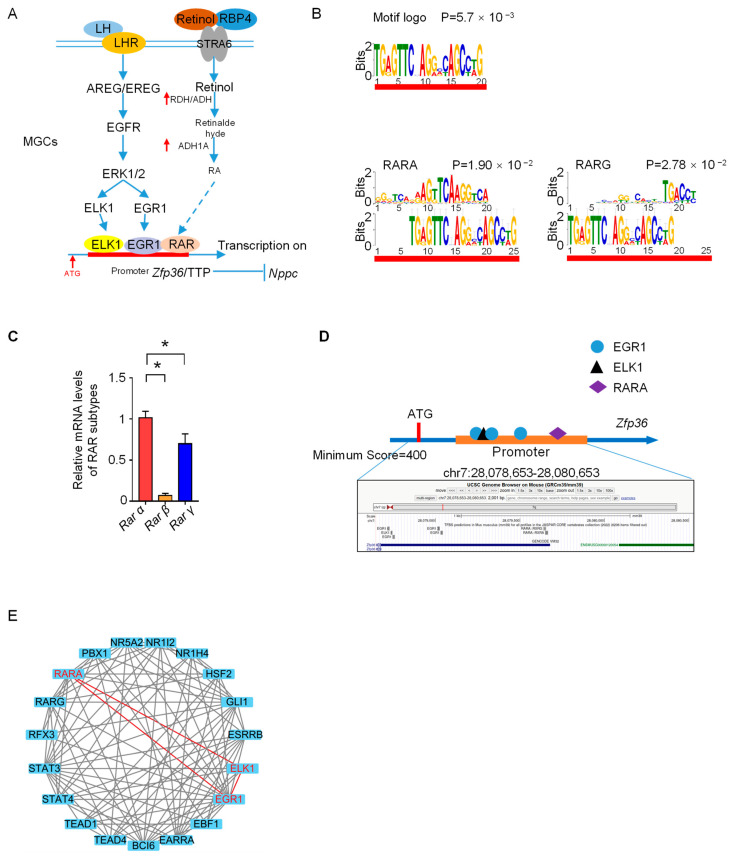
RAR is a potential transcription factor of *Zfp36*. (**A**) Schematic illustration of proposed parallel pathways that underlie the Zfp36 upregulation in MGCs in response to LH surge. (**B**) The most significant motifs based on comprehensive motif analysis on sequences located in the promoter regions of Zfp36 using MEME (v5.5.1). The identified motif was compared against a database of known mouse motifs named HOCOMOCO Mouse (v11 Core). The RAR-related DNA binding motifs were: RARA and RARG. (**C**) The relative mRNA levels of RAR subtypes in preovulatory MGCs. * *p* < 0.05. (**D**) Transcription factors EGR1 and ELK1 were predicted by the JASPAR2022 TFBS mm39 collection, which represented separate tracks in mouse genome assembly chr7:28,078,653-28,080,653. (**E**) STRING Protein-Protein Interaction (PPI) analysis of the transcription factors predicted by the MEME (v5.5.1). Red lines showed the interaction between the EGR1, ELK1 and RARA.

Given MGC is critical to mediating RA-prompted oocyte meiotic resumption, we next examined the underlying mechanism in MGCs. Our recently published study showed that LH-induced rapid up-regulation of *Zfp36* in MGCs. *Zfp36* encodes an mRNA-destabilizing protein TTP that degrades “meiosis arrester” *Nppc* mRNA and, in turn, regulates oocyte meiotic resumption [19]. The spatiotemporal coincidence of LH-induced upregulation of RA signaling and *Zfp36* in MGCs (Figure 4A), as well as their functional role in prompting meiotic resumption, led us to ask if RA signaling may participate in Zfp36 upregulation. Supporting this hypothesis, the bioinformatic analysis showed TGAGTTCNAGGVCAGCCWG motif fitting the binding sequence for RARα and RARγ within the promoter region of Zfp36 (Figure 4B), in line with their expression patterns in preovulatory MGCs (Figure 4C). We noticed RAR motifs were located proximal to the binding sequences of EGR1 and ELK1, two identified transactional factors of *Zfp36* in a previous study [35] (Figure 4D), and reconstructed the protein-protein network of potential transcription factors also implied the interaction between EGR1, ELK1 and RAR (Figure 4E).

### 3.5. RA Prompts the Expression of Zfp36 in MGC via RAR

**Figure 5:**
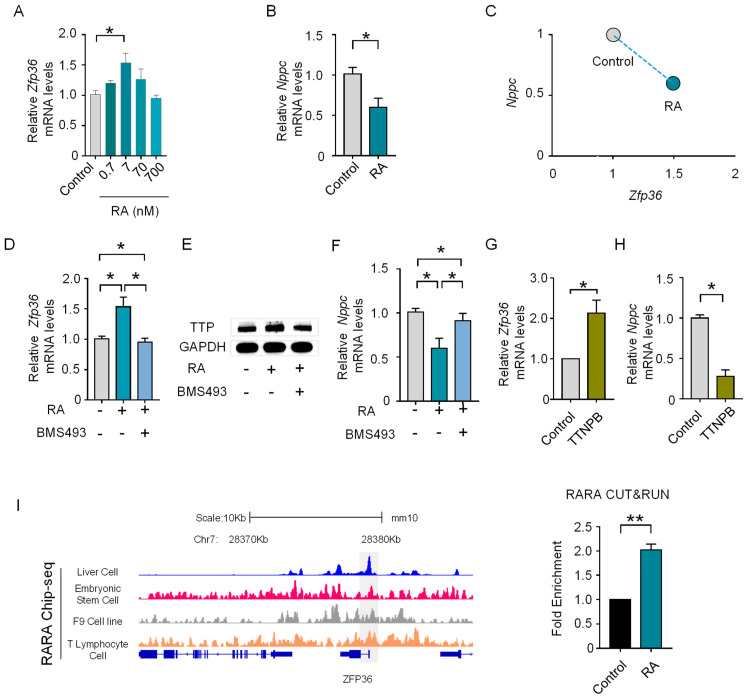
RA promotes the expression of *Zfp36* in MGC via RAR. (**A**) The mRNA levels of *Zfp36* in MGCs cultured with or without different concentrations of RA. * *p* < 0.05 (**B**) The mRNA levels of *Nppc* in MGCs cultured with or without 7 nM RA. * *p* < 0.05 (**C**) The negative correlation between *Zfp36* and *Nppc* expression levels in MGCs cultured with or without 7 nM RA. (**D**) The mRNA levels of *Zfp36* in MGCs cultured with RA alone or together with 1 μM BMS493. * *p* < 0.05 (**E**) The Western blotting analysis of TTP in MGCs cultured with RA alone or together with BMS493. * *p* < 0.05 (**F**) The mRNA levels of *Nppc* in MGCs cultured with RA alone or together with 1 μM BMS493. * *p* < 0.05 (**G**,**H**) The mRNA levels of *Zfp36* (**F**) and *Nppc* (**G**) in MGCs cultured with or without 0.1 μM TTNPB. * *p* < 0.05 (**I**) The IGV screenshots (left) of examples of RARA Chip-seq analysis in different cells collected by the Cistrome data browser and the RARA binding sites are highlighted by the grey area. The right panel showed the RARA CUT&RUN results by detecting the highlighted area. ** *p* < 0.01.

To directly test if RA participates in LH-induced rapid upregulation of *Zfp36* in MGCs, we next isolated preovulatory MGCs and supplemented RA at different concentrations during in vitro culture. Results showed that a 7 nM RA supplementation medium could significantly upregulate *Zfp36* (Figure 5A) and led to a significant decrease in *Nppc* mRNA levels (Figure 5B), showing a negative correlation with those in the control group (Figure 5C). Furthermore, the stimulating effect of RA on *Zfp36* expression (Figure 5D and Figure S1C) can be eliminated by RAR inhibition, highlighting the essential role of RAR in mediating these effects. The role of RA in inducing Zfp36 upregulation and the mediating role of RAR was also confirmed at the protein levels (Figure 5E), as well as the corresponding inhibitory effect on *Nppc* mRNA levels (Figure 5F). To further confirmed the critical mediating role of RAR, we showed the RAR-specific agonist TTNPB could recapitulate the effect of RA supplementation on *Zfp36* upregulation (Figure 5G and Figure S1D) and *Nppc* decrease (Figure 5H). Finally, Cut&Run analysis provided direct evidence that RAR binding on the *Zfp36* promoter region was significantly increased in response to RA exposure (Figure 5I).

### 3.6. RA Signaling Cooperates with Parallel EGF Signaling to Upregulate Zfp36 through an ERK-Dependent Pathway

**Figure 6:**
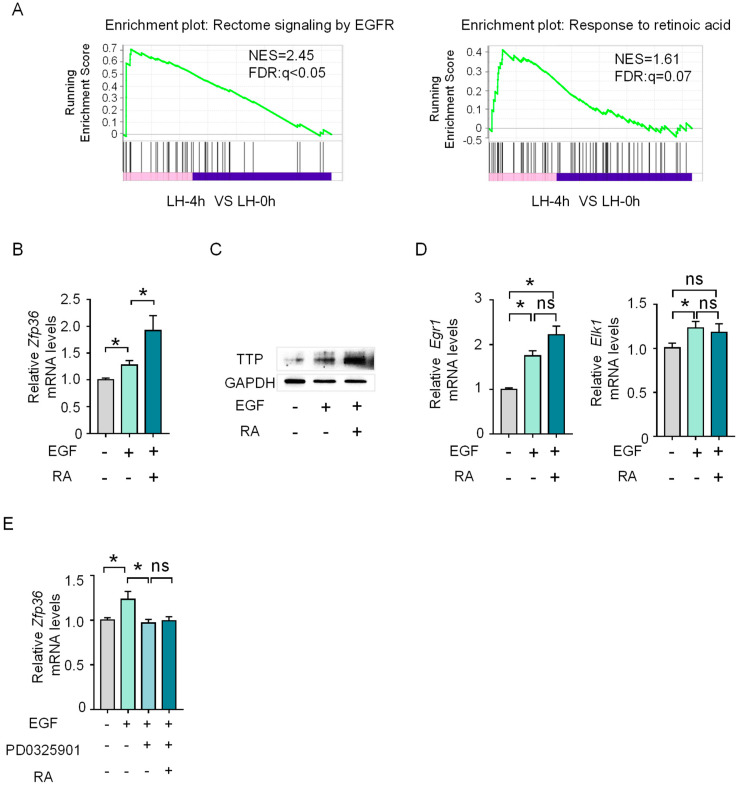
RA signaling cooperates with parallel EGF signaling to upregulate Zfp36 through the ERK-dependent pathway. (**A**) GSEA analysis showing enrichment of Rectome signaling by EGFR and Response to retinoic acid, Normalized enrichment score (NES) and false discovery rate (FDR) q value are shown. (**B**) The mRNA expression levels of *Zfp36* in MGCs cultured with EGF alone or together with RA. * *p* < 0.05 (**C**) The Western blotting analysis of TTP in MGCs cultured with EGF alone or together with RA. * *p* < 0.05 (**D**) The mRNA expression levels of *Egr1* and *Elk1* in MGCs cultured with EGF alone or together with RA. * *p* < 0.05, ns, not significant (*p* > 0.05). (**E**) The mRNA expression levels of *Zfp36* in MGCs cultured with EGF alone or various combinations with RA and PD0325901. * *p* < 0.05, ns, not significant (*p* > 0.05).

Given our previous study has reported that EGF signaling is also the critical mediator of LH-induced upregulation of Zpf36 and oocyte meiotic resumption [19], and By reanalyzing a previously published transcriptome that presented rapid effects of LH on gene expression in the mural granulosa cells (MGCs) of preovulatory mouse follicles [20], our GSEA analysis showed that both EGFR signaling and RA signaling was activated in preovulatory MGCs in response to LH (Figure 6A). The spatiotemporal coincidence led us to examine the relationship between these two signalings in the regulation *Zfp36* upregulation. Using in vitro cultured preovulatory MGC model, we found RA supplementation further increased EGF-induced upregulation of *Zfp36* (Figure 6B), which was also confirmed on the protein level (Figure 6C). However, RA supplementation did not increase the expression levels of EGF signaling-dependent transcriptional factors *Elk1* and *Egr1* (Figure 6D), suggesting EGF and RA signalings may cooperate and are in parallel with each other to upregulate Zfp36. Moreover, given we have previously reported LH activates Zfp36 through the EGFR-ERK1/2–Dependent Pathway [19], we next wanted to know if the synergistic effect between RA and EGF on Zfp36 upregulation also depends on the ERK1/2 pathway. We found that when the ERK pathway was blocked by PD0325901, RA could not further enhance *Zfp36* upregulation in the presence of EGF, implying the dependence on the ERK pathway (Figure 6E).

## 4. Discussion

It has been clear that RA regulates myriads of physiological functions and processes. Among these, RA acts as a key driver of entry into meiosis in both male and female germ cells [36]. In contrast, however, whether RA regulates the exit from oocyte meiotic arrest has not been definitely determined, despite the presence of some suggestive evidence. Results based on an in vitro study showed RA enhanced mouse oocyte maturation in vitro and improved fertilization and development rates in a dose-dependent manner [37]. In addition, exogenous injections of VA into gilts after the second estrous cycle dramatically enhanced the GVBD rate of oocytes [38].

**Figure 7:**
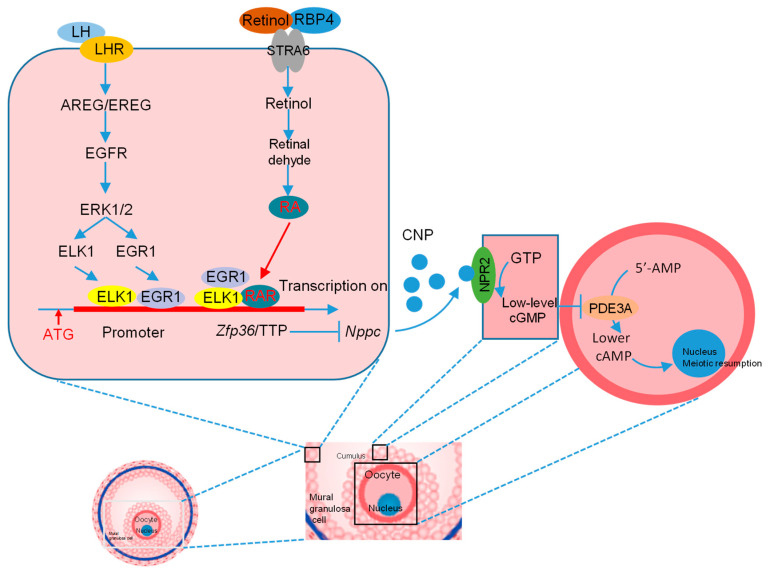
Working model illustrating the mechanism that underlies the role of RA in regulating LH-induced oocyte meiotic resumption. The left indicates the previously reported pathway responsible for Zfp36 upregulation, and the right indicates the pathway identified in the present study.

In the present study, using well-established in vivo and in vitro models, we identify that RA plays a critical role in ensuring successful oocyte meiotic resumption in an MGC-dependent manner (Figure 7). Of note, although it has been frequently reported in previous studies [39], our study further highlights the importance of MGCs in mediating LH-induced oocyte resumption. We proved, in vitro and in vivo, that MGCs are indispensable for ensuring the prompting effect of RA on oocyte meiotic resumption.

Our study has also focused on the mechanism underlying the role of RA in prompting oocyte meiotic resumption. Current knowledge mainly focused on the LH-induced signaling cascades in MGCs, e.g., LHR and EGFR activation, CNP-NPR2 inactivation and decrease in cGMP [13,40], while our study showed a remarkable metabolic change in MGCs and its functional role in regulating oocyte meiotic resumption. The spatiotemporal coincidence of LH-induced EGF signaling and RA signaling in MGCs also suggests the synergistic cooperation between these two signalings. We also found RA signaling acts in parallel with EGF signaling. This is in line with the previous notion that both EGF-dependent and -independent intrafollicular pathways are important for inducing oocyte meiotic resumption [21,41].

With regards to the receptor that mediates the prompting effect of RA signaling on meiotic resumption, our experiments based on both atRA supplementation and receptor inhibition suggested RAR is the main receptor. In general, it is well documented RAR is the preferred receptor for all-trans retinoic acid (atRA), and RXR is preferred for 9-cis RA. There are three subtypes of RAR, i.e., Rarα, Rarβ and Rarγ, and our bioinformatic analysis showed the binding motifs of RARα and RARγ within the promoter region of Zfp36, suggesting that both RARα and RARγ may participate in the transcriptional regulation of Zfp36.

The present study, together with our previous work [19], indicates that Zfp36 is the co-target of these two signalings. *Zfp36* encodes tristetraprolin (TTP), a ubiquitously expressed mRNA-destabilizing protein. We have reported that Zfp36/TTP is one of the regulatory components responsible for the LH-induced rapid decrease in *Nppc* mRNA/CNP, a critical prerequisite for oocyte meiotic resumption. We have also revealed the mechanism underlying the transient upregulation of Zfp36 in response to the LH. ELK1 and EGR1 are the essential transcriptional factors that are responsible for the LH-induced rapid upregulation of Zfp36, and their function is dependent on the EGF-ERK pathway [42]. In the present study, we have coupled RA signaling to EGF signaling and proposed that the transcriptional factors in these two parallel pathways may cooperate together to upregulate Zfp36 in response to LH.

In summary, our data provide direct evidence that intrafollicular RA signaling is important for LH-induced oocyte meiotic resumption. In response to LH surge, RA signaling is activated in MGCs. We also identify that RA signaling participates in LH-induced Zpf36 via RAR, which may cooperate with ELK1 and EGR1. Our results not only advance current knowledge of LH-induced Zfp36 upregulation and Nppc decrease but also further emphasize again the importance of MGCs in regulating oocyte meiotic resumption. In addition, these findings extend our understanding of the role of RA in regulating oocyte meiosis: it not only governs the initiation of oocyte meiosis during female embryonic development but also participates in the resumption of oocyte meiotic arrest when LH restarts meiosis.

## Data Availability

The data presented in this study are available on request from the corresponding author.

## Supplementary Materials

The following supporting information can be downloaded at: https://www.mdpi.com/article/10.3390/genes14040946/s1, Figure S1: Screening experiments of suitable concentration for RA, RAR antagonist (BMS493), RAR agonist (TTNPB) in follicile culture or MGCs culture; Table S1: All the primers used in this research; Table S2: Vitamin A deficient diet ingredients table.
