# Supplementary material for: Intrafollicular Retinoic Acid Signaling Is Important for Luteinizing Hormone-Induced Oocyte Meiotic Resumption
Source: Genes (Basel). 2023 Apr 20;14(4):946. doi: 10.3390/genes14040946 (PMC10137601; doi:10.3390/genes14040946)
Supplement: Supplementary file 1 [file genes-14-00946-s001.zip › genes-2281111-supplementary.pdf]

Supplementary Materials

Intrafollicular Retinoic Acid Signaling Is Important for Luteinizing Hormone-Induced Oocyte Meiotic Resumption

Fupeng Wang †, Yawen Tang †, Yijie Cai, Ran Yang, Zongyu Wang, Xiaodong Wang, Qianying Yang, Wenjing Wang, Jianhui Tian and Lei An *

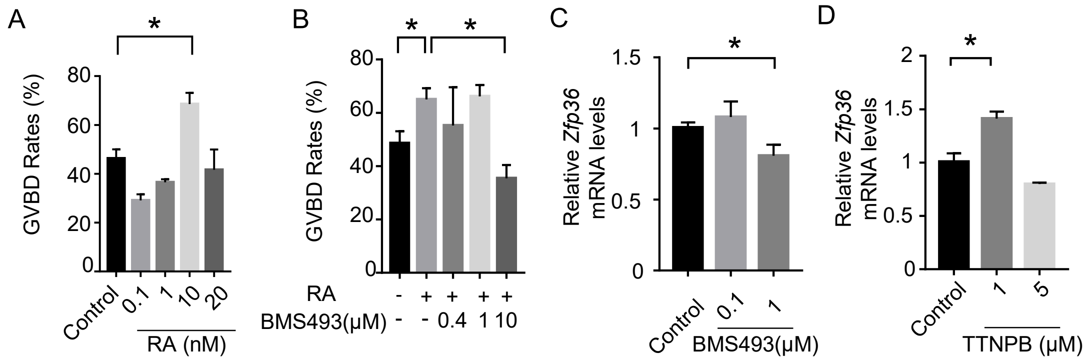

Figure S1. Screening experiments of suitable concentration for RA, RAR antagonist (BMS493), RAR agonist (TTNPB) in follicile culture or MGCs culture. (A) The GVBD rates of oocytes collected from preovulatory follicles cultured with different concentration of RA. *$P < 0.05$. (B) The GVBD rates of oocytes collected from preovulatory follicles cultured with RA alone or together with different concentration of BMS493. *$P < 0.05$. (C) The mRNA expression levels of *Zfp36* in MGCs cultured with different concentration of BMS493. *$P < 0.05$. (D) The mRNA expression levels of *Zfp36* in MGCs cultured with different concentration of TTNPB. *$P < 0.05$.

Table S1. All the primers used in this research.

Gene	Forward primer	Reverse primer
qPCR primers		
Gapdh	CCTGGAGAAACCTGCCAAGTAT	GGAAGAGTGGGAGTTGCTGTTG
Nppc	GGTCTGGGATGTTAGTGCAGCTA	TAAAAGCCACATTGCGTTGGA
Zfp36	CGAGAGCCTCCAGTCGATGAG	GGATGGAGTCCGAGTTTATGTTCC
Egr1	CCGAGCGAACAACCCTATGA	TGGGATAACTCGTCTCCACCAT
Elk1	ATCCCTGCTCCCCACACATAC	CCACTGGACGGAAACTGGAA
Areg	GCAGATACATCGAGAACCTGGAG	CCTTGTCATCCTCGCTGTGAGT
Ereg	TGCTTTGTCTAGGTTCCCACC	GGCGGTACAGTTATCCTCGG
Btc	TTCGTGGTGGACGAGCAAACTC	CCATGACCACTATCAAGCAGACC
CUT&RUN qPCR primer		
RARA-*Zfp36*	GTCCCGGAAGCTCTAGTGG	GACTGTCGGTTCGCAGAAGT

Table S2. Vitamin A deficient diet ingredients table

Vitamin A deficient diet	
Ingredients	Weight (g/kg)
V1002 (Vitamins and minerals)	10
Casein	200
Cystine	3
Starch	397
Maltodextrin	132
Saccharose	100
Cellulose	50
Soya-bean oil	70
TBHQ	0.014
M1003G	35
Choline Bitartrate	2.5
V1002 Ingredients table	
Ingredients	Weight (g/kg)
Vitamin A (50%)	0
Vitamin D3	0.2
Vitamin E	10.08
Vitamin K	0.078
Biotin	0.1
Vitamin B12	0.25
Folic acid	0.21
Nicotinic acid	3
Calcium pantothenate	1.6
Vitamin B6	0.7
Vitamin B2	0.75
Vitamin B1	0.6
Sucrose	982.5
